# Premenstrual symptoms across the lifespan in an international sample: data from a mobile application

**DOI:** 10.1007/s00737-022-01261-5

**Published:** 2022-08-26

**Authors:** Liisa Hantsoo, Shivani Rangaswamy, Kristin Voegtline, Rodion Salimgaraev, Liudmila Zhaunova, Jennifer L. Payne

**Affiliations:** 1 Department of Psychiatry and Behavioral Sciences, Johns Hopkins University School of Medicine, Baltimore, MD USA; 2 Johns Hopkins University School of Medicine, Baltimore, MD USA; 3 Department of Pediatrics, Johns Hopkins University School of Medicine, Baltimore, MD USA; 4 Flo Health UK Limited, London, UK; 5 Department of Psychiatry and Neurobehavioral Sciences, University of Virginia, Charlottesville, VA USA

**Keywords:** Menstrual cycle, Premenstrual syndrome (PMS), Premenstrual dysphoric disorder (PMDD), Premenstrual exacerbation, Perimenopause

## Abstract

Premenstrual symptoms, including physical and mood symptoms, affect a large proportion of women worldwide. Data on premenstrual symptoms across nations and age groups is limited. In the present study, we leveraged a large international dataset to explore patterns in premenstrual symptom frequency with age. A survey was administered to users of the Flo mobile application (app), aged 18 to 55. The survey queried app users about a range of premenstrual symptoms. Respondents were asked whether they experienced each symptom every menstrual cycle, some cycles, or never. Age was also captured and categorized as 18–27, 28–37, 38–47, 48–55. Data was summarized and Pearson’s chi square test for count data assessed differences in symptom frequency by age group. A sample of 238,114 app users from 140 countries responded to the survey. The most common symptoms reported were food cravings (85.28%), mood swings or anxiety (64.18%), and fatigue (57.3%). Absentmindedness, low libido, sleep changes, gastrointestinal symptoms, weight gain, headaches, sweating or hot flashes, fatigue, hair changes, rashes, and swelling were significantly more frequent with increasing age (*p*’s < 0.001). Mood swings and anxiety did not vary by age group. Of the respondents, 28.61% reported that premenstrual symptoms interfered with their everyday life each menstrual cycle. In a large international sample, the majority of women reported premenstrual food cravings, mood changes, and fatigue every menstrual cycle. Mood symptoms did not vary by age group, suggesting that premenstrual mood changes are a persistent issue among women of reproductive age.

**Supplementary Information:**

The online version contains supplementary material available at 10.1007/s00737-022-01261-5.

## Introduction

Premenstrual symptoms affect individuals across nations and cultures. Approximately 80% of women report experiencing at least one mood or physical symptom premenstrually (Schoep et al. 2019). However, there is limited data on the occurrence of premenstrual symptoms across nations and by different age groups. Premenstrual symptoms can be physical, such as fatigue, irritability, bloating, or breast tenderness, or affective, such as irritability, anxiety, or mood lability, and occur on a continuum of severity (Angst et al. 2001; Borenstein et al. 2003, 2005). Premenstrual symptoms occur during the late luteal phase of the menstrual cycle (roughly the week before menses onset), resolve within a few days of menstruation onset, and are absent in the postmenstrual week (Epperson et al. 2012; Halbreich 2004). Premenstrual syndrome (PMS) includes mild to moderate physical and/or mood symptoms and occurs in roughly 20% of women, while premenstrual dysphoric disorder (PMDD), at the severe end of the continuum, includes more impairing symptoms and is found in 3 to 8% of women (Epperson et al. 2012; Halbreich et al. 2003; Wittchen et al. 2002; Yang et al. 2008). Premenstrual symptomatology in both PMS and PMDD is thought to be due to an abnormal sensitivity to steroid hormones that fluctuate across the menstrual cycle (Schmidt et al. 1998; Martinez et al. 2016; Hantsoo and Epperson 2020; Schweizer-Schubert et al. 2021). For the purposes of this paper, we will refer to the general term “premenstrual symptoms” when it is not clear that symptoms meet criteria for either PMS or PMDD, and only use the terms PMS or PMDD when criteria are met.

Premenstrual symptoms may occur at any point during the female reproductive years, from menarche to menopause. PMS or PMDD may initiate in the teen years (Rapkin and Mikacich 2013), but most often initiate in a woman’s 20 s (Strine et al. 2005). Premenstrual symptoms may peak around age 35 (Dennerstein et al. 2011a; Tschudin et al. 2010), although few studies have examined the prevalence of premenstrual symptoms across age groups. A large study of over 3500 women reported the prevalence of moderate to severe mood-based PMS as 10.7% in women aged 15–24, 8.6% in women aged 25–34, 11.2% in women aged 35–44, and 10.8% in women aged 45–54 (Tschudin et al. 2010), suggesting that women in mid-life were slightly more likely to experience moderate to severe premenstrual mood symptoms than younger or older groups. However, another cross-sectional study found that premenstrual symptoms were less common in women ages 36 to 44 years, compared with younger women (Freeman et al. 1995). It is also possible that certain symptoms worsen or improve with age. For instance, some symptoms may worsen in the mid-thirties (Dennerstein et al. 2011b) but improve prior to the menopause transition (Dennerstein et al. 2011a), although longitudinal data is lacking. One goal of the present study was to examine the frequency of a range of premenstrual symptoms in those aged 18 to 55, accounting for a wide age range.

Premenstrual symptoms occur across cultures and nationalities. In a meta-analysis assessing the prevalence of PMS in twelve countries, the highest prevalence was reported in Iran (98%) and the lowest prevalence was reported in France (12%) (Direkvand-Moghadam et al. 2014). European countries in general reported a lower prevalence of PMS compared to Asian countries included in the study. Reports of premenstrual symptom severity also vary by country. Individuals in the UK, Brazil, Japan, Korea, and Australia reported relatively greater severity and duration of premenstrual symptoms, while those in Hong Kong and Pakistan reported the lowest severity and duration of symptoms (Dennerstein et al. 2011b).

Finally, premenstrual symptoms may have a significant impact on an individual’s daily functioning (Halbreich et al. 2003). In a study of over 4000 women from 19 different countries, those with moderate to severe premenstrual symptoms showed increased absenteeism and decreased productivity at work (Heinemann et al. 2012). In a survey of over 40,000 women, 38% were unable to perform activities of daily life due to premenstrual symptoms (Schoep et al. 2019). Among adolescents, at least 20% reported PMS symptoms associated with functional impairment (Rapkin and Mikacich 2013). There is minimal evidence on how functional impairment varies by country. One study reported there was no decrease in work productivity in Asian countries among those with PMS/PMDD, while all other continents found work-related decreased productivity in those with PMS/PMDD (Heinemann et al. 2012). Another study assessing European and Latin American countries found that impairment in function was most severe in the home setting, followed by social, educational, and work settings, which was consistent across both continents (Dennerstein et al. 2010). Given the impairment caused by premenstrual symptoms, assessing and properly treating premenstrual symptoms is an important clinical and public health issue.

The aims of the present study were to leverage a large international dataset, collected via a mobile application (app) Flo, to (1) explore prevalence of premenstrual symptoms across ages ranging from 18 to 55 and (2) explore prevalence of premenstrual symptoms by nation.

## Materials and methods

### a. Overview

This was a cross-sectional survey study of women aged 18 to 55. The survey was administered to users of the Flo Health mobile app, a free app available on both the Android and iOS platforms. The survey was administered from May 12, 2017 to June 23, 2020 using a chatbot dialogue. Participating women who answered the “Premenstrual Syndrome (PMS) Survey” were included. The survey was released in 10 languages (English, Russian, Spanish, German, French, Italian, Portuguese, Polish, Japanese, and Chinese) and was available to all women using the Flo app. The geographical location of the user was estimated based on the IP address. All participants in the study agreed to the use of their deidentified and aggregated data for research purposes. Data were deidentified and sent in aggregate to the Johns Hopkins research team. The Johns Hopkins Institutional Review Board (IRB) reviewed the protocol and acknowledged the application as a minimal risk secondary research protocol.

### b. Survey

Survey questions addressed a range of physical and cognitive-affective premenstrual symptoms. These included mood swings or anxiety; absentmindedness; sleep changes; headaches; hot flashes or sweating; fatigue; breast soreness, swelling, or hypersensitivity; constipation or diarrhea; food craving; weight gain; increased appetite; swelling; abdominal spasms; low libido; changes in hair; or skin rashes. Participants were also asked if premenstrual symptoms interfered with their daily life. For each item, participants could respond “Yes, every cycle,” “Yes, but not every cycle,” “No,” or “Prefer not to answer.” Specific questions are listed in the supplementary Online Resource 1.

### c. Statistical analyses

Summary data was available by age group within the sample; individual level data was not available due to international data privacy guidelines. Data was examined descriptively by country. To examine age effects, data was categorized into four age groups: 18–27, 28–37, 38–47, and 48–55 years old. Pearson’s chi-squared test for count data with independent samples was conducted to test differences in symptom frequency by age group, considering the frequency of the response “Yes, every cycle” for each item. A significant omnibus chi-square test statistic indicated a group difference in the symptom by age. The specific age strata where symptoms were more or less prevalent than in the total sample were probed by post hoc evaluation of residuals. Post hoc tests were based on adjusted Pearson residuals that measured the difference between observed and expected cell counts, with observed difference from expected inferred from Bonferroni-adjusted *p* values to account for multiple comparisons. Post hoc *p* values tested the deviation from the expected prevalence in the total sample in a specific age group, with the null assumption that prevalence in each age group was equal to prevalence in the total sample. R and R Studio were used to conduct analyses (R Core Team 2020).

## Results

### a. Participant characteristics

Data was obtained from mobile app users across 140 countries. Only countries with at least 500 respondents were included. This resulted in data from *n* = 238,114 unique users who contributed data to these analyses. This sample included 162,390 (68.14%) in the 18–27 years age group; 61,210 (25.69%) in the 28–37 years age group; 13,684 (5.74%) in the 38–47 years age group, and 1026 (0.43%) in the 48–55 years age group.

### b. Frequency of premenstrual symptoms

**Fig. 1:**
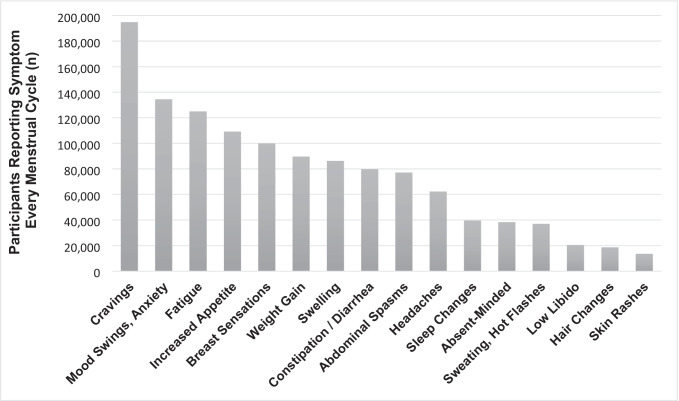
Frequency of premenstrual symptoms reported by survey respondents. Numbers reflect participants who reported experiencing the symptom every menstrual cycle

Across the sample of 238,114 users, the most common symptom that participants reported experiencing every cycle was food cravings (85.28%), followed by mood swings or anxiety (64.18%) and fatigue (57.3%) (Table 1). All other symptoms were experienced by fewer than 50% of the responders. Among respondents, 28.61% reported that premenstrual symptoms interfered with their everyday life each cycle, and an additional 34.84% reported that premenstrual symptoms interfered with their everyday life some cycles. Percentages are based on number of participants that responded to an individual question, which may be fewer than the total sample *n*; frequency of all symptoms across the full sample is shown in Fig. 1.

**Table 1:** Premenstrual symptom frequency by age group

Symptom	Total		18–27		28–37		38–47		48–55		*p* value
	*n*	%	*n*	%	*n*	%	*n*	%	*n*	%	
Cravings	194,755	85.28	131,380	84.46	50,944	86.84	11,565	87.78	866	87.56	< 2.2e − 16*
Mood swings, anxiety	134,485	64.18	91,160	64.09	34,840	64.47	7,908	64.12	577	61.65	0.1569
Fatigue	125,018	57.30	80,602	54.37	35,558	63.26	8,237	64.64	621	64.82	< 2.2e − 16*
Appetite increase	109,245	48.40	71,117	46.31	30,674	52.81	6,941	53	513	52.45	< 2.2e − 16*
Breast sensations	99,963	46.14	66,110	44.91	26,903	48.2	6,478	51.13	472	49.42	< 2.2e − 16*
Weight gain	89,740	39.04	53,090	33.9	28,589	48.44	7,465	56.36	596	60.14	< 2.2e − 16*
Swelling	86,197	38.62	52,197	34.4	26,536	46.13	6,923	53.31	541	55.72	< 2.2e − 16*
Constipation/diarrhea	79,794	36.00	51,588	34.24	22,624	39.6	5,182	40.13	400	41.28	< 2.2e − 16*
Abdominal spasms	77,067	33.26	53,477	33.87	19,175	32.24	4,143	31.09	272	27.23	< 2.2e − 16*
Headaches	62,260	28.91	40,807	27.9	16,788	30.25	4,324	34.29	341	35.89	< 2.2e − 16*
Sleep changes	39,587	18.77	25,155	17.58	11,052	20.31	3,093	24.92	287	30.56	< 2.2e − 16*
Absentminded	38,282	18.09	23,704	16.51	11,318	20.72	2,989	24	271	28.83	< 2.2e − 16*
Sweating, hot flashes	37,017	17.28	24,130	16.59	9,866	17.87	2,773	22.09	248	26.11	< 2.2e − 16*
Low libido	20,566	9.66	10,623	7.35	7,659	13.94	2,099	16.79	185	19.56	< 2.2e − 16*
Hair changes	18,696	8.53	11,770	7.9	5,376	9.53	1,411	11.03	139	14.46	< 2.2e − 16*
Skin rashes	13,659	6.20	8,120	5.42	4,482	7.89	986	7.67	71	7.37	< 2.2e − 16*
Interference	59,782	28.61	39,546	27.87	15,993	29.71	3,970	32.31	273	29.17	

Number (%) of participants reporting symptoms every menstrual cycle by age group. *P* values < 0.05 were considered significant (*). *P* values refer to differences between the age groups

### c. Frequency of premenstrual symptoms by age group

We examined the frequency of symptoms in each age group (Table 1). Symptom frequency was significantly greater than expected with increasing age for most symptoms. That is, symptoms were more common in the older age groups than expected if symptoms were equivalent by age. These included absentmindedness (*X*^*2*^ (3, *N* = 211,577) = 864.3, *p* < 0.0001), low libido (*X*^*2*^ (3, *N* = 213,000) = 2877.8, *p* < 0.0001), sleep changes (*X*^*2*^ (3, *N* = 210,880) = 611.92, *p* < 0.0001), gastrointestinal symptoms (*X*^*2*^ (3, *N* = 221,667) = 631.53, *p* < 0.0001), weight gain (*X*^*2*^ (3, *N* = 229,850) = 5783.4, *p* < 0.0001), headaches (*X*^*2*^ (3, *N* = 215,342) = 321.44, *p* < 0.0001), sweating or hot flashes (*X*^*2*^ (3, *N* = 214,158) = 316.49, *p* < 0.0001), fatigue (*X*^*2*^ (3, *N* = 218,169) = 1640.3, *p* < 0.0001), hair changes (*X*^*2*^ (3, *N* = 219,114) = 292.41, *p* < 0.0001), rashes (*X*^*2*^ (3, *N* = 220,470) = 2839.2, *p* < 0.0001), and swelling (*X*^*2*^ (3, *N* = 223,213) = 3812, *p* < 0.0001). In particular, these symptoms were less frequent than expected in the 18–27-year-old age group and more frequent than expected in the 28–37-, 38–47-, and 48–55-year-old age groups.

Frequency of abdominal spasms was significantly lower than expected as age increased (*X*^*2*^ (3, *N* = 231,705) = 98.503, *p* < 0.0001). Specifically, abdominal spasms were more frequent than expected in the 18–27-year-old age group and less frequent than expected in the 28–37-, 38–47-, and 48–55-year-old age groups.

Other symptoms were less frequent than expected in the 18–27 age group, more frequent than expected in the 28–37 and 38–47 age groups, and not significantly different than expected for the 48–55 age group. These symptoms were food cravings (*X*^*2*^ (3, *N* = 228,383) = 267.63, *p* < 0.0001), breast sensations (*X*^*2*^ (3, *N* = 216,633) = 314.17, *p* < 0.0001), and increased appetite (*X*^*2*^ (3, *N* = 225,718) = 836.23, *p* < 0.0001).

Mood swings and anxiety did not differ significantly by age group (*X*^*2*^ (3, *N* = 209,556) = 5.2119, *p* = 0.1569).

### d. Frequency of premenstrual symptoms by country

**Fig. 2:**
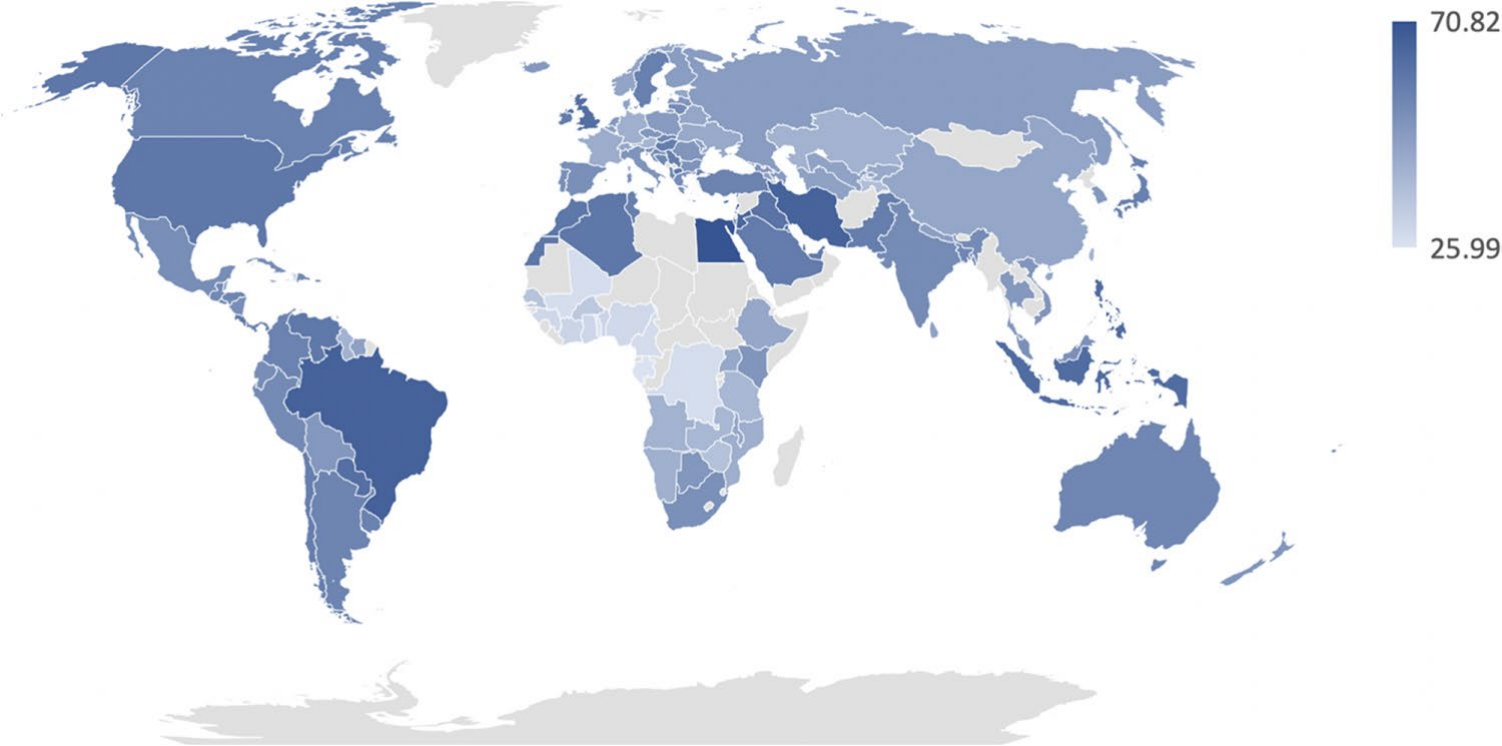
Percentages reflect participants within each country who reported experiencing premenstrual mood or anxiety symptoms every menstrual cycle, with darker shades representing greater percentages

**Fig. 3:**
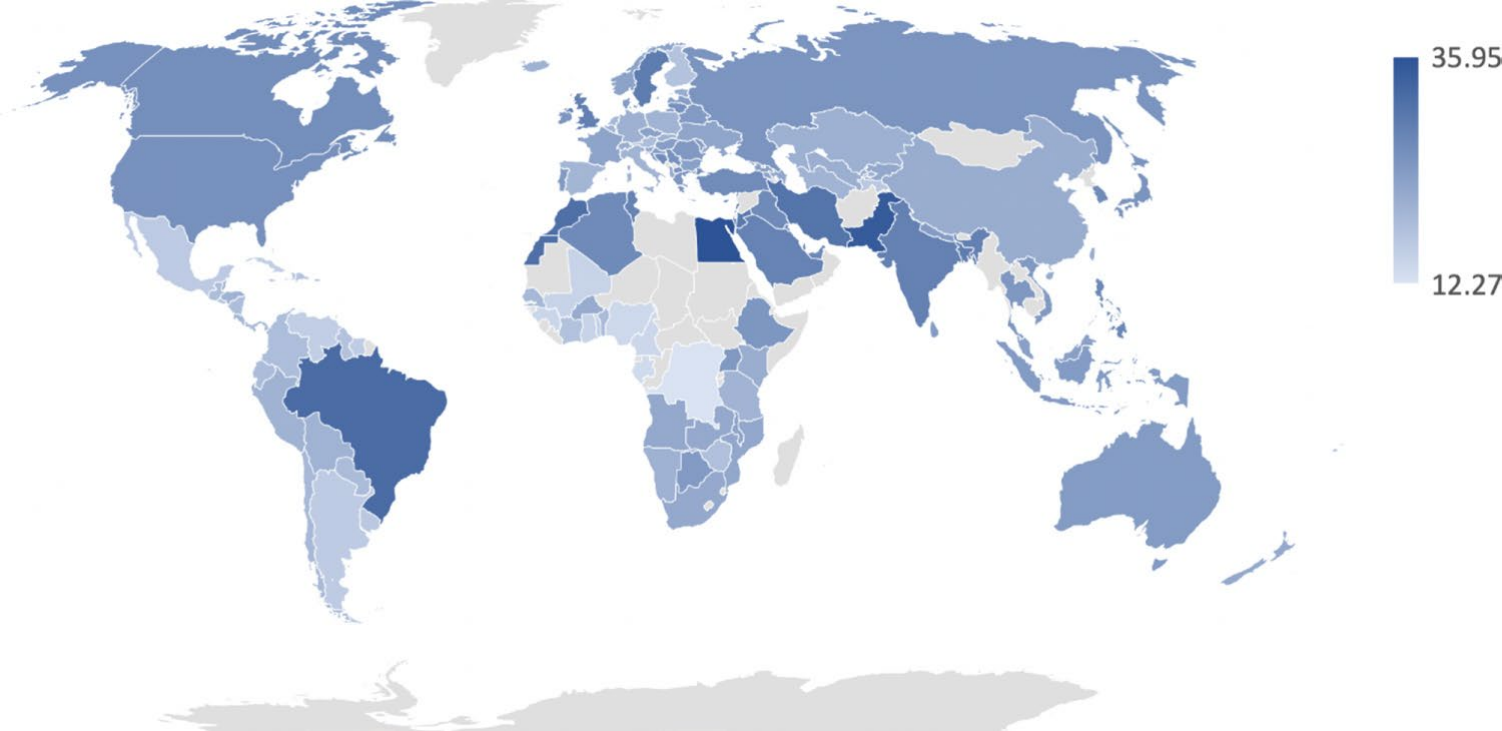
Percentages of participants within each country who reported interference from premenstrual symptoms every menstrual cycle, with darker shades representing greater percentages

We examined rates of premenstrual mood and anxiety symptoms among the 140 countries (Fig. 2). Premenstrual mood symptoms reported every cycle were most common in Egypt (70.82%), Lebanon (67.42%), and Brazil (66.60%) and least common in Mali (27.64%), Gabon (26.02%), and Togo (25.99%). Participants across nations also reported that premenstrual symptoms caused interference in their functioning every cycle (Fig. 3). Participants reported interference most frequently in Egypt (68.59%), Pakistan (61.53%), and Brazil (64.73%) and least frequently in Nigeria (40.65%), Gabon (33.84%), and Congo (35.17%).

## Discussion and conclusions

Within a large international sample of 238,114 adults experiencing menstrual cycles, the majority reported experiencing premenstrual food cravings, mood swings or anxiety, and fatigue. Furthermore, while physical symptoms tended to increase with age, mood swings and anxiety were reported at similar frequencies across age groups.

Our results were similar to that of Schoep and colleagues, who in 2019 published a study on the premenstrual symptoms among 42,879 women in the Netherlands (Schoep et al. 2019). Schoep et al. found that the most common premenstrual symptoms were dysmenorrhea (~ 85%), psychological complaints (77%), and tiredness (71%) (Schoep et al. 2019). In our study, psychological complaints (64.18%) and tiredness or fatigue (57.3%) were also among the most common symptoms, although less frequent than in Schoep et al.’s study. Also, we found food cravings (85.28%), not dysmenorrhea, to be the most commonly reported symptom. Together, our results align with those of Schoep et al. to suggest that premenstrual psychological complaints are a key symptom experienced by individuals worldwide. This is important information for clinicians in primary care, gynecology, or mental health settings who work with individuals experiencing menstrual cycles. Clinicians should be aware that food cravings, mood symptoms, and fatigue are common premenstrually, and that premenstrual mood symptoms may occur across age ranges while physical symptoms may be more common in older menstruators. This is particularly important when evaluating individuals for mental health concerns, as premenstrual mood symptoms may be mistaken for other types of mood disorders and vice versa.

Participants in our sample also reported significant interference with daily activities due to their premenstrual symptoms. Among respondents who reported on interference, 63% reported that premenstrual symptoms interfered with their everyday life either every cycle or some cycles. The 28.61% who reported that premenstrual symptoms interfered with functioning every cycle was similar to the results of Schoep et al., who found that 38.4% of women reported interference with function during their period. This is critical, as women with PMS report reduced productivity and more missed days at work as well as increased frequency of healthcare utilization (Borenstein et al. 2003).

Regarding changes in symptoms with age, we found that symptom frequency increased with age for absentmindedness, low libido, sleep changes, gastrointestinal symptoms, weight gain, headaches, sweating or hot flashes, fatigue, hair changes, and swelling. Conversely, frequency of abdominal spasms decreased with age. Findings on worsening of premenstrual symptoms with age have been mixed. Some studies suggest that premenstrual symptoms lessen with age (Freeman et al. 1995, 2004; Silva et al. 2008; Sternfeld et al. 2002). For instance, a cross-sectional study found that premenstrual symptoms improved with age, with women ages 36 to 44 being less symptomatic than those 20 to 35 years old (Freeman et al. 1995). However, other cross-sectional data on PMS symptoms in women between 15 and 54 years of age found that women aged 35 to 44 had the greatest symptom severity relative to other age groups (Tschudin et al. 2010). Other studies suggest that certain symptoms worsen with age, particularly somatic symptoms (Sylvén et al. 2013). Another study found an inverted U-shaped relationship between symptom severity and age, with premenstrual syndrome severity peaking at middle age (Dennerstein et al. 2011b). Some data report that the relationship between PMS and age vary depending on the particular symptom (Dennerstein et al. 2011a). In that study, the symptoms demonstrating an inverted U-shaped severity curve with the peak at 35 years of age included both physical (skin disorders, fatigue, appetite changes, body aches, abdominal bloating, swelling of extremities, breast tenderness, weight gain) and affective (anger, depressed mood, irritability). However, in that study, confusion, mood swings, and abdominal cramps decreased with age, and other symptoms had no relationship with age. Longitudinal research is needed to more fully characterize which premenstrual symptoms may worsen, or improve, over time.

In the current sample, many of the symptoms that increased with age are associated with perimenopause, such as hot flashes, absentmindedness, and low libido. Thus, it makes sense that these premenstrual symptoms increased with age, potentially as women were approaching menopause, given that the sample included women up to age 55. The mean age of menopause (final menstrual period) is 51 years in the USA (Menopause - Symptoms and causes. 2021). Our finding that abdominal spasms decreased with age is similar to findings by Dennerstein and colleagues, in which abdominal cramps decreased with age (Dennerstein et al. 2011a).

Intriguingly, mood swings and anxiety did not differ significantly by age group. Indeed, across age groups, 61–64% of respondents reported mood or anxiety symptoms every menstrual cycle. This represents the majority of participants in this international sample across age ranges, suggesting that premenstrual mood symptoms are a key public health issue globally. Some studies suggest that premenstrual mood symptoms are less common among women ages 45 years and older (Strine et al. 2005), or lessen with age (Freeman et al. 1995, 2004; Silva et al. 2008; Sternfeld et al. 2002). However, in the present sample, our data suggests that premenstrual mood symptoms are a common, persistent complaint across age ranges. This high prevalence is important information for clinicians, as some may be unaware of the commonness of premenstrual mood complaints, leaving premenstrual mood symptoms underrecognized and undertreated (Hantsoo et al. 2022).

Finally, the prevalence of mood swings and anxiety symptoms varied widely between countries. Premenstrual mood and anxiety symptoms were most common in Egypt, Lebanon, Brazil, Iran, and Jordan, with 64% or more participants reporting premenstrual affective symptoms every menstrual cycle. Premenstrual affective symptoms were least common in Cameroon, Congo, Mali, Gabon, and Togo, with fewer than 28% of participants reporting monthly mood changes. This is similar to a meta-analysis that found wide variation in prevalence of PMS across twelve nations, ranging from 12% (France) to 98% (Iran) (Direkvand-Moghadam et al. 2014). We also found wide variation in percentage of participants reporting that premenstrual symptoms affected their functioning, from 35.17 to 68.59%. This is novel, as there is little evidence to date on how functional impairment varies by country. One study reported less impact of premenstrual symptoms on work productivity in Asian countries compared to non-Asian countries (Heinemann et al. 2012), while another found that impairment was similar between European and Latin American countries (Dennerstein et al. 2010). As we did not query about specific impacts of premenstrual symptoms on functioning (e.g., missed days of work), but asked more broadly, “Do PMS symptoms interfere with your everyday life?” this may have produced the broad range of results, as respondents may have interpreted the question of interference differently.

In sum, a majority of participants in this large international sample reported premenstrual food cravings, fatigue, and mood changes, with the frequency of physical symptoms increasing with age and mood symptoms occurring uniformly across age groups. Although additional research is needed before this information can be implemented in clinical settings, our data does suggest that premenstrual symptoms are common, and clinicians should be aware of this. In addition, longitudinal research is required to understand how premenstrual physical and mood symptoms progress across the lifetime, including reproductive transitions such as the postpartum or perimenopause.

This study’s main strength was its large, international sample encompassing women across a range of geographic areas and ages. There were also several limitations. One limitation of the study is that it was based on aggregated, pooled data due to international mobile app data privacy rules, i.e., individual level data was not available. This prevented us from examining potential health or demographic mediators, such as parity or hormonal contraceptive use, on reported premenstrual symptoms. The use of aggregated data also prevented us from examining whether premenstrual symptoms may have been affected by medication use (e.g., antidepressants) or medical or psychiatric comorbidities. Furthermore, the data format precluded us from statistically testing for differences in premenstrual symptoms between countries. The data were collected from users of a mobile app, meaning that all participants needed access to a mobile telephone, as well as wireless internet or cellular internet access, which may have selected for a well-resourced sample. Another important limitation was that the data was self-report and was not based on clinician rating or validated scales designed specifically to measure premenstrual symptoms in a clinical sample. Thus, it is possible that participants over-reported or under-reported premenstrual symptoms. Indeed, this study was designed to examine the presence of premenstrual symptoms, not their severity. Relatedly, it is possible that participants interpreted the question of symptom interference differently between respondents, as described above. Other studies have used more objective measures of premenstrual symptom interference, such as days missed from work (Halbreich et al. 2003; Yang et al. 2008; Heinemann et al. 2012; Dennerstein et al. 2010). There is also the possibility of reporting bias — it may be that individuals who are more aware of premenstrual symptoms are more likely to use a mobile app to track symptoms, or are more likely to respond to a survey about premenstrual symptoms.

In summary, we found that in a large international sample, premenstrual mood symptoms were common and occurred at similar frequencies across age groups. In contrast, physical premenstrual symptoms tended to increase with age. Finally, we found that a significant proportion of women reported that premenstrual symptoms interfered with daily functioning every cycle in the overall sample but that this proportion varied widely by country, indicating a need for further study.

## Supplementary Information

Below is the link to the electronic supplementary material.

Supplementary file1 (DOCX 105 KB)

Supplementary file2 (DOCX 58.1 KB)

## Declarations

This study involved analysis of aggregated data. The Johns Hopkins Institutional Review Board (IRB) reviewed the protocol as Not Human Subjects Research (NHSR). The IRB determined that the protocol did not constitute human subjects research under DHHS or FDA regulations and acknowledged the application as a minimal risk secondary research protocol.
