# Supplementary material for: Premenstrual symptoms across the lifespan in an international sample: data from a mobile application
Source: Arch Womens Ment Health. 2022 Aug 26;25(5):903–10. doi: 10.1007/s00737-022-01261-5 (PMC9492621; doi:10.1007/s00737-022-01261-5)
Supplement: Supplementary file 1 — Supplementary file1 (DOCX 105 KB) [file 737_2022_1261_MOESM1_ESM.pdf]

Supplementary Table

Premenstrual Symptom Interference by Country

COUNTRY	% Reporting Premenstrual Symptom Interference Every Cycle
Egypt	35.95
Pakistan	34.66
Brazil	31.96
Morocco	31.22
Iran	30.48
Jordan	29.52
Bangladesh	29.15
Sweden	29.15
UK	28.47
India	28.39
Lebanon	28.32
Tunisia	28.1
Saudi Arabia	28.05
Qatar	27.9
United Arab Emirates	27.19
Philippines	27.18
Iraq	26.85
Algeria	26.76
Brunei Darussalam	26.61
Korea	26.61
Turkey	26.59
Bosnia and Herzegovina	26.55
Israel	26.19
Canada	26.1
Kuwait	25.99
US	25.76
Lithuania	25.65
Serbia	25.56
Japan	25.35
Cyprus	25.34
Macedonia	25.2
Russian Federation	25.13
Greece	24.9
Malta	24.86
Ethiopia	24.81
Ireland	24.8
Thailand	24.57
Uganda	24.51

Romania	24.42
Viet Nam	24.36
Portugal	24.23
Botswana	24.17
Belarus	24.14
Australia	24.03
Indonesia	23.97
Nepal	23.95
Sri Lanka	23.89
Mauritius	23.84
Denmark	23.71
Moldova	23.65
Hungary	23.44
Latvia	23.43
Croatia	23.3
Malaysia	23.27
Bulgaria	23.15
Georgia	23.15
Maldives	23.03
Czech Republic	22.86
Norway	22.73
France	22.63
Malawi	22.62
Armenia	22.51
Réunion	22.44
Ukraine	22.35
Azerbaijan	22.12
Mozambique	22.01
Slovenia	22.01
Kyrgyzstan	21.93
Angola	21.79
South Africa	21.75
Zambia	21.71
New Zealand	21.69
Andorra	21.36
Burkina Faso	21.29
China	21.17
Estonia	21.06
Albania	21.03
Switzerland	21.01
Kenya	20.96

Taiwan	20.87
Uzbekistan	20.77
Kazakhstan	20.52
Singapore	20.44
Germany	20.4
Namibia	20.3
Peru	20.19
Honduras	20.18
Iceland	20.17
Bolivia	20.1
Slovakia	20.1
Belgium	20.02
Poland	19.98
Trinidad and Tobago	19.96
Barbados	19.91
Cabo Verde	19.91
Tanzania	19.9
Turkmenistan	19.9
Belize	19.88
Italy	19.88
Guadeloupe	19.76
Spain	19.68
Costa Rica	19.62
Netherlands	19.22
Puerto Rico	19.13
Senegal	19.13
Chile	19.07
Hong Kong	19
Panama	18.92
Paraguay	18.61
Benin	18.6
Ecuador	18.44
Austria	18.36
Guatemala	18.35
Nicaragua	18.33
Colombia	18.3
El Salvador	17.97
Zimbabwe	17.87
Côte d'Ivoire	17.66
Guyana	17.52
Finland	17.47

Jamaica	17.44
Dominican Republic	17.2
Haiti	16.83
Tajikistan	16.63
Uruguay	16.57
Fiji	16.56
Bahamas	16.42
Mexico	16.22
Argentina	16.03
Suriname	15.91
Cuba	15.65
Venezuela	15.64
Togo	15.48
Ghana	14.8
Mali	14.74
Guinea	14.29
Cameroon	14.11
Nigeria	13.88
Gabon	13.48
Congo	12.27

Percentage of participants reporting premenstrual symptoms causing interference in daily functioning every menstrual cycle by country.
