# Supplementary material for: Premenstrual symptoms across the lifespan in an international sample: data from a mobile application
Source: Arch Womens Ment Health. 2022 Aug 26;25(5):903–10. doi: 10.1007/s00737-022-01261-5 (PMC9492621; doi:10.1007/s00737-022-01261-5)
Supplement: Supplementary file 2 — Supplementary file2 (DOCX 58.1 KB) [file 737_2022_1261_MOESM2_ESM.pdf]

**APPENDIX**

**Survey Questions**

1. Do you experience mood swings or increased anxiety before your period?
2. Are you particularly absent-minded shortly before your period?
3. Do your sleep patterns change before your period?
4. Do you experience headaches shortly before menstruation?
5. Do you experience sweating, hot flashes, or chills before menstruation?
6. Do you feel fatigue before your period?
7. Do you feel unusual sensations in your breasts (soreness, nipple swelling, hypersensitivity) before menstruation?
8. Do you have constipation or diarrhea before your period?
9. Do you crave certain foods before your period?
10. Do you notice any weight gain about a week before your period?
11. Do you observe an increased appetite shortly before menstruation?
12. Do you observe swelling (abdomen, limbs) before your period?
13. Do you suffer from spasms (dragging pains in the lower abdomen) a few days before your period?
14. Do you have low libido before your menstruation?
15. Do you notice any changes in your hair (whether it gets greasier, more brittle, etc.) shortly before your period?
16. Do you suffer from skin rashes before your period?
17. Do PMS symptoms interfere with your everyday life?
